# Handgrip exercise by the non-affected hand increases venous return in the contralateral axillary vein in patients with stroke: a pilot study

**DOI:** 10.1186/s13104-018-3475-6

**Published:** 2018-06-08

**Authors:** Hiroyuki Hayashi, Motoyuki Abe, Bunzo Matsuoka

**Affiliations:** Faculty of Care and Rehabilitation, Seijoh University, 2-172 Fukinodai, Tokai, Aichi 476-8588 Japan

**Keywords:** Stroke, Venous return, Upper limb, Rhythmic resistance exercise, Affected hand, Ultrasound

## Abstract

**Objective:**

Treatment of hand edema is important for maintaining upper limb function in patients with stroke, although the effects of many such treatments have been limited. This study aimed to examine, using ultrasound, the effect of handgrip exercise by the non-affected hand of stroke patients on venous return in the affected upper limb.

**Results:**

Seven men participated, within 6 months of a unilateral first-ever stroke. With the patient supine, examinations were performed on the axillary vein of the affected side. The diameter and flow velocity of the axillary vein on the affected side were measured during two regimens: at rest or during rhythmic resistance exercise (30% of maximum grip strength for 20 s) performed by the non-affected hand. The venous flow volume in the axillary vein was then calculated using the data obtained. During resistance exercise by the non-affected hand, there were significant increases in both venous flow velocity (p = 0.01, *d* = − 0.80) and volume (p = 0.01, *d* = − 0.74) on the affected side, compared with baseline. The present preliminary study found that rhythmic resistance exercise with the non-affected hand increased venous flow velocity and volume in the affected upper limb of patients with stroke.

## Introduction

Edema or swelling of an affected hand is often observed in patients with post-stroke hemiplegia. The incidence of hand edema varies from 9 to 80%, depending on the study’s definitions and measurements [1–3]. One of the causes of hand edema is obstructed venous return [4]. Post-stroke edema has been attributed to impaired functioning of the venous return and lymphatic system as a result of immobility [5]. Persistent hand edema is associated with pain and fibrotic tissue [6], resulting in an unfavorable effect on hand function. These conditions may even lead to permanent loss of upper limb function. Therefore, the treatment to reduce hand edema is important for maintaining upper limb function in patients with stroke.

In previous studies, treatments for alleviating edema included intermittent compression [7], upper limb elevation [6], wrist orthosis [8], continuous passive motion [9], and the use of kinesio tape [10], but their effects were limited. It therefore became apparent that novel, more effective interventions for post-stroke hand edema were required. Recently, Ishii et al. [11] reported that the deoxy-hemoglobin of non-exercising upper limb muscles in 13 healthy men decreased during contralateral one-armed cranking exercise. The direction of changes in deoxy-hemoglobin is determined by changes in the venous blood volume [12]. Hence, Ishii et al.’s results suggested that venous return might be promoted in the affected hand by contralateral hand exercise.

Ultrasound (US) can provide information about the diameter and flow velocity in the deep veins of the arm [13]. However, there have been no previous reports on US evaluation of venous return in the affected hands of stroke patients.

We hypothesized that contralateral venous blood volume increases according to non-affected hand exercise and can be measured by US. The purpose of the present study was to examine, using US, the effect of handgrip exercise by the non-affected hand on the venous return in the affected upper limb in patients with stroke. The present study’s results could have consequences for the development of a new intervention for post-stroke hand edema based on venous return.

## Main text

### Methods

#### Subjects

Seven men (mean age 60 years, range 44–73 years) within 6 months of their unilateral first-ever stroke participated in the study. All patients were admitted to the post-acute rehabilitation unit of Gifu Central Hospital or Tokai Memorial Hospital in Japan. Patients with severe cognitive impairment and those with cardiac pacemakers were not included in the study. The study protocol, which met the standards of the Declaration of Helsinki, was approved by the research ethics committees of Seijoh University (Approval Number 2016C0022), Gifu Central (Approval Number 133), and Tokai Memorial Hospital. Informed written consent was obtained from each participant before all procedures. All experiments were performed in a soundproof room in which the temperature was maintained at 24–26 °C.

#### US measurements

All examinations were performed on the axillary vein of the affected side with the patient in supine position and the arms kept alongside the body. The diameter and flow velocity of the axillary vein in the affected upper limb was measured using a Xario US device (Toshiba Medical Systems, Tokyo, Japan) with a 7.5-MHz linear probe. Accurate identification of the vein was confirmed by a positive compressive pressure test and by assessing venous flow via color Doppler imaging.

The diameter (mm) of the axillary vein of the affected upper limb was assessed using B-mode images of the vessel in the longitudinal plane. Time-averaged mean velocity (cm/s) was also assessed using pulse-wave Doppler imaging.

Doppler spectra for calculating the time-averaged mean velocity (cm/s) was obtained in a longitudinal plane at the same site with an insonating angle maintained at < 60°. The sample volume was positioned at the center of the vessel, and the amplitude was adjusted to allow sampling of 50–70% of the vessel lumen [13].

Using the obtained data, we calculated the venous flow volume (ml/min) in the axillary vein using the following equation:

$$ {\text{Venous}}\,{\text{flow}}\,{\text{volume}}\left( {{\text{ml}}/\hbox{min} } \right) = {\text{radius}}\left( {\text{mm}} \right) \times {\text{radius}}\left( {\text{mm}} \right) \times\uppi \, \left( {{\text{circular}}\,{\text{constant}}} \right) \times {\text{time}} - {\text{averaged}}\,{\text{mean}}\,{\text{velocity}}\left( {{\text{cm}}/{\text{s}}} \right) \times 60/100. $$

#### Procedure

A digital handgrip dynamometer (Takei Scientific Instruments Co., Ltd., Niigata, Japan) was used to measure the maximum grip strength of the non-affected hand of each patient. Grip strength (kg) was measured and recorded as the average of two repetitions. Following the measurement of grip strength, participants were allowed rest breaks of at least 10 min in supine position.

The diameter and flow velocity of the axillary vein on the affected side were measured during two distinct regimens: baseline (rest) or rhythmic resistance exercise (30% of maximum grip strength for 20 s with the movement guided by a metronome at a pace of 1 s/cycle). After a rest period, the diameter (mm) and time-averaged mean velocity (cm/s) were measured as baseline data. Following these measurements, the same parameters were measured during rhythmic resistance exercise. During the handgrip exercise, subjects were told to maintain normal breathing. Each US measurement lasted 15 s.

Blood pressure and heart rate were measured at baseline and immediately after the rhythmic resistance exercise regimen using an automatic monitor (HEM-780; Omron, Kyoto, Japan).

#### Statistical analysis

Paired t-tests were performed to detect the significance of the differences between the baseline values and those measured during the rhythmic resistance exercise for the dynamometer readings, time-averaged mean velocity, and venous flow volume in the axillary vein; blood pressure; and heart rate. In addition, the effect sizes (Cohen’s *d*) were calculated between the two regimens. All statistical analyses were performed with HAD version 16 software [14]. A value of p < 0.05 was considered to indicate statistical significance. The effect size was calculated with Cohen’s “*d*” method, where *d* = 0.2 indicated a small effect, *d* = 0.5 a medium effect, and *d* = 0.8 a large effect.

### Results

The baseline and rhythmic resistance data are shown in Table 1. There was no significant difference in the venous diameters between the baseline and rhythmic resistance exercise values. In contrast, there was a significant increase in the time-averaged mean velocity during resistance exercise compared with baseline. There was also a significant increase in the venous flow volume during resistance exercise compared with baseline. For the time-averaged mean velocity and venous flow volume, there was not only a significant difference between baseline and resistance exercise but the effect size was moderate.

**Table 1:** Venous hemodynamic measurements using ultrasound at baseline and during resistance exercise

Measurement	Baseline	Resistance exercise	p	*d*
Venous diameter (mm)	6.70 ± 2.72	6.82 ± 2.64	0.50	− 0.05
Time-averaged mean velocity (cm/s)	2.66 ± 1.23	3.99 ± 2.05	0.01	− 0.80
Venous flow volume (ml/min)	52.35 ± 31.59	77.82 ± 38.15	0.01	− 0.74
Systolic blood pressure (mmHg)	120.86 ± 14.10	126.00 ± 13.66	0.10	− 0.37
Diastolic blood pressure (mmHg)	73.29 ± 6.85	74.29 ± 8.92	0.62	− 0.13
Heart rate (beats/min)	66.86 ± 9.10	66.86 ± 7.10	> 0.99	0.00

Data are expressed as mean ± standard deviation

There was no significant difference in the blood pressure or heart rate between the baseline and rhythmic resistance exercise measurements. No fatigue or adverse events (e.g., pain, discomfort) were reported by the participants throughout the study.

### Discussion

In the present pilot study, we examined whether handgrip exercise by the non-affected hand of a stroke patient promotes venous return in the contralateral, affected arm. We calculated venous flow volume according to the venous diameter and the time-averaged mean flow velocity in the axillary vein measured by US. We found that rhythmic resistance exercise by the non-affected hand significantly increased venous flow velocity and volume in the affected upper limb, although the diameter of the axillary vein showed no statistically significant increase. Additionally, a moderate effect was observed for a difference in both venous flow velocity and volume between the measurements at baseline and during resistance exercise. These new findings suggest that exercise by the non-affected hand promotes venous return in the contralateral, affected upper limb in hemiplegic patients.

This study is the first report of increased venous return due to exercise by the contralateral hand without any influence of the systemic circulation. As peripheral venous return is to the heart, it is recognized in two main factors: the muscle pump and the respiratory pump. In the present study, we speculated on the possibility of a third pump—a “contralateral limb exercise pump”—although the mechanism remains unclear. We suggest that it might be an effect of the pressure distribution of the superior vena cava during the exercise cycle.

There was no significant difference in the venous diameter between the baseline and resistance exercise conditions in this study, but the venous flow velocity significantly increased. In a previous study, Ojima et al. [15] reported no differences in the popliteal vein or common femoral vein diameters between the at-rest condition and during electrical muscle stimulation, although peak venous flow velocities were higher during electrical muscle stimulation than at rest. Likewise, the increased venous flow volume resulted primarily from increases in the time-averaged mean flow velocity in this study using rhythmic handgrip exercise. Ishii et al. [11] reported a heart rate increase during the early period (10–20 s) of voluntary one-arm cranking with 35–40% maximum voluntary contraction (MVC). Victor et al. [16] reported that the mean arterial pressure and heart rate increased during the first minute of rhythmic handgrip at 30% MVC. However, there were no statistically significant differences in blood pressure or heart rate between the baseline and resistance exercise measurements in the present study because of the short exercise duration (20 s). Because of the differences in the exercise (including contraction and exercise times), the blood pressure and heart rate must not have been influenced in the present study. There were no adverse events attributable to the study conditions. We believe that the protocol used in the present study is safe.

Following stroke, hand edema on the affected side is a common complication. In previous studies, the effects of treatment for edema has had limited effectiveness, indicating that a novel treatment for edema of the affected hand would be required. In this pilot study, we clarified that venous return in the affected arm was promoted by handgrip exercising with the non-affected hand and that it was safe. Although using the affected arm for any activity is generally difficult for patients with hemiplegia, it is easy for them to move the non-affected hand. Hence, further research is necessary to confirm and expand upon the results of this study.

### Conclusion

This preliminary study of seven male stroke patients found that resistance exercise by the non-affected hand increased venous flow velocity and volume in their contralateral, affected upper limb. The study included only a small number of subjects in supine position. Additional research is therefore encouraged to confirm the effectiveness of exercise by the non-affected hand to increase the venous return of the affected upper limb while in a sitting position or using a handgrip exercise without resistance.

## Limitations

The present study has some limitations. First, because it was a pilot study using a small sample, the results should be considered preliminary and viewed with caution, although resistance exercise induced a significant increase in venous flow velocity and volume compared with baseline. Second, resistance exercise was set at only 30% MVC of the handgrip. Ishii et al. [11] suggested the possibility that one-armed passive arm motion causes a change in venous blood oxygenation and volume in non-contracting arm muscles. Therefore, the use of handgrip exercise without resistance should be established in a future study. Third, we measured the hemodynamics of the venous circulation in supine patients with stroke. It is generally recommended, however, that stroke patients be mobilized to an active sitting or standing position as soon after stroke as possible. Stern et al. [17] reported that the venous volume of upper-extremity edema differs depending on the patient’s posture. Therefore, venous hemodynamics must be measured in stroke patients not only in the supine position but also when sitting.

## Abbreviations

US: ultrasound
MVC: maximum voluntary contraction
